# Backgrounding steers on temperate grasses mixed with vetch and/or using energy supplementation

**DOI:** 10.5713/ajas.18.0603

**Published:** 2018-11-27

**Authors:** Eduardo Felipe Colerauz de Oliveira Lazzarotto, Luís Fernando Glasenapp de Menezes, Wagner Paris, Marcos Luis Molinete, Gean Rodrigo Schmitz, José Henrique Ignacio Baraviera, Roberta Farenzena, Adalberto Luiz de Paula

**Affiliations:** 1 Faculty of Animal Science, Universidade Tecnológica Federal do Paraná, Dois Vizinhos, PR 85660-000, Brazil

**Keywords:** Forage Allowance, Pasture Mixed, Grazing Time, Feeding Behavior, Legume Pasture

## Abstract

**Objective:**

The aim was to evaluate backgrounding beef steers on oat + ryegrass pastures mixed with vetch and/or using energy supplementation.

**Methods:**

A randomized block design with three treatments and three replications was used. The treatments were: grass + supplement (oat + ryegrass + supplementation), legume + supplement (oat + ryegrass + vetch + supplementation) and grass + legume (oat + ryegrass + vetch). A continuous grazing system with a variable stocking rate was used. Twenty-seven intact crossbred steers (1/4 Marchigiana, 1/4 Aberdeen Angus and 2/4 Nellore) aged 7 months old and average weight of 190 kg were used. Steers were supplemented at 1% of the body weight of ground corn. The experiment lasted 84 days, between May and August 2014. Behavioral assessments were performed two times per experimental period, for 24 hours.

**Results:**

The forage mass was different between treatments, being greater for steers fed without legume. The accumulation rate, forage allowance, and stocking rate did not differ between treatments due to the adequate adjustment of forage allowance. The final weight of animals, as well as the dry matter intake (kg/d), did not differ between treatments. However, forage intake was higher for non-supplemented animals in relation to supplemented steers. Supplement intake did not alter the total digestible nutrient intake due to pasture quality. Animals fed grass + supplement had higher live weight gain per area than those fed grass + legume. Animals without supplementation spent more time in grazing.

**Conclusion:**

Feeding behavior was not altered by mixing with vetch or supplementation. Non-supplemented animals started the grazing peak earlier and spent more time in grazing than those supplemented; however, the average daily gain was similar between treatments. The live weight gain per hectare was 47% higher in pastures in which the animals received supplementation compared with those mixed with vetch, a consequence of the substitutive effect.

## INTRODUCTION

Beef cattle production under grazing systems allows a more competitive cost in the beef industry in meeting the world demand for food, besides being a sustainable economic approach. The use of temperate pastures is considered a key technology for intensification of grass-based productive systems. Among these technologies to intensify production, we can highlight energy supplementation, nitrogen fertilization or the mixing of grasses with legumes [1,2]. The responses associated with temperate grasses promoted by these technologies are diverse and depend on the soil-plant-animal interaction.

Fertilization is a technique heavily questioned due to economic and environmental issues. As an alternative, mixing grasses with legumes has aroused interest, allowing extended grazing periods and increased diet quality for livestock production [1]. The black oat (*Avena strigosa* Schreb.) and ryegrass (*Lolium multiflorum* Lam.) mixed with vetch (*Vicia sativa* L.) are species of good nutritional quality, with high digestibility and high content of degradable nitrogen (N), which can be lost and excreted via urine if it is not adequately supplied in the diet.

A mixture of grasses with legume improves the supply of protein, which is already high due to the presence of temperate grasses. However, this protein is lost in the rumen, limiting an increase in protein reaching the intestine [3]. To minimize these losses, the use of energy supplementation in those pastures with higher protein content can be a strategy. Energy supplementation is a widespread technique that increases pasture carrying capacity and the gain per area. Besides improving the efficiency of N use in grazing systems, energy supplementation provides great quantities of non-structural carbohydrates to the rumen, leading to improved livestock performance [4]. However, this technology must be used with extreme precision due to its high cost.

Supplementation is an alternative to reduce protein loss, which can reach 30% to 40% [5]. The dietary increase in energy via supplement (carbohydrate) reduces losses and makes the system more efficient, consequently reducing age at slaughtering in beef production. Therefore, this study aimed to evaluate the mixed of temperate grasses with legume and/or the use of energy supplementation in backgrounding steers.

## MATERIALS AND METHODS

### Local and ethics committee

**Figure 1:**
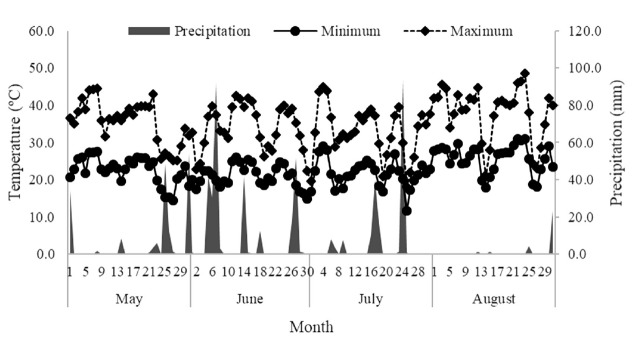
Rainfall and temperature (maximum and minimum) in Dois Vizinhos, Paraná, from May to August 2014, GEBIOMET (2014).

The study was conducted at the Universidade Tecnológica Federal do Paraná –Dois Vizinhos campus, PR, Brazil. The climate of the region is the subtropical humid mesotherm (Cfa) according to the classification of Köppen. The climate and rainfall data during the experimental period were obtained at the automatic meteorological station of the campus, located about 100 meters from the experimental area (Figure 1). The study was conducted according to the rules of the Animal Use Ethics Committee (CEUA, Comissão de Ética no Uso de Animais), under the protocol no. 2013-008 of the Universidade Tecnológica Federal do Paraná.

### Experimental design

A randomized block design with three treatments and three replications was used. The treatments evaluated were: grass + supplement (oats + ryegrass + supplementation), legume + supplement (oats + ryegrass + vetch + supplementation) and grass + legume (oats + ryegrass + vetch), with three replications. Supplementation of ground corn plus 1% mineral salt was given daily at 11 h at 1% of body weight.

### Experimental period, pasture management, and animal’s description

The experiment lasted 100 days, between May and August 2014. The animals were adapted during 16 days before the evaluation period (84 days). Three 28-day periods were considered for the evaluations. In each period, forage and animal traits were assessed (animal performance and behavior).

Seven hectares were used for the study, subdivided into nine paddocks of 0.78 ha each. Forage sources were: black oat (*Avena strigosa* Schreb.) cv. EMBRAPA BRS 139, ryegrass (*Lolium multiflorum* Lam.) cv. Fepagro São Gabriel and vetch (*Vicia sativa* L.) cv. Amethyst. The following seed densities were used: 60 kg of black oat, 30 kg of ryegrass and 30 kg of vetch. The basal fertilization was performed with 350 kg/ha of formulated 05-20-10 (N-P-K) fertilizer. Urea was used to provide N (100 kg/ha), divided into three equal applications. Ryegrass was sown, then oats and vetches were planted using a no-till system, spaced 17 cm apart to a depth of 3 to 5 cm.

Twenty-seven non-castrated crossbred steers (1/4 Marchigiana, 1/4 Aberdeen Angus, and 2/4 Nellore) with an initial age of 7±2 months and average weight of 190 kg were used, like as three rumen-fistulated Holstein steers, for the forage intake evaluation.

### Forage adjustment

Forage mass (FM) was estimated by the double-sampling method [6], using a square 0.25 m^2^ for 20 visual evaluations (5 cuttings) of the pasture. The cuttings were done to ground level. The forage accumulation rate per day was measured using two exclusion cages per paddock. The cages were positioned at representative points of the average sward height, with similar mass and morphological composition. The forage masses, inside and outside the cage, were obtained within the 0.25 m^2^ square, by cutting to the ground level. This evaluation was performed every 28 days. After each cutting, the cages were moved to other points of the paddocks, following the same methodology. The forage accumulation (dry matter [DM] kg/ha) was obtained by the difference between the forage mass inside the cage in the current period and outside the cage in the previous period. To estimate the forage accumulation rate per day (kg/ha/d), the total was divided by the number of days in each period.

### Stocking adjustment

A continuous grazing system with variable stocking rates (SR) was used, using non-tester animals of the same age and genetic group to adjust the SR through the “put-and-take” technique [7]. The SR was estimated every 28 days using a forage allowance of 9 kg DM/100 kg live weight (LW) throughout the period, keeping three tester animals per paddock.

The average SR for the grazing period, expressed in kg/ha, was calculated by the sum of the average weight of tester animals + the average weight of each non-tester animal, multiplying the result by the number of grazing days and then dividing the result by the total number of grazing days. The forage allowance was estimated using the methodology, defined as the direct relation of forage mass shared by the SR [8].

### Animal performance

Average daily weight gain (ADG) was calculated by the difference between the final and initial weight of tester animals, in each experimental period, divided by the number of grazing days. Before each weighing, the animals were fasted from solids and liquids for 14 hours. The live weight gain per hectare per day (LWG/ha/d) was obtained by multiplying the average weight gain of tester animals by the number of days and the number of animals per hectare in each period.

### Intake dry matter

Relative to dry matter intake (DMI) estimation, an external marker was used (chromium dioxide, CrO_2_). The marker was weighed on an analytical scale, wrapped in paper, and then 10 g were introduced directly into the rumen of the fistulated animals. This process was performed daily for 12 days. The marker was administered for 12 days and feces were collected in the last five days. A double Latin square design 3×3 (three treatments and three replications) was used. The total DMI was estimated using the equation: DMI = FP×(1–IVDMD). In this equation, FP is the fecal production (kg), which is a result of the relationship between the concentration of chromium supplied (already known) and concentration of chromium in feces, obtained in laboratory by atomic absorption spectrophotometry [9]. The IVDMD, in turn, is the *in vitro* dry matter digestibility (g/kg) [10]. Forage dry matter intake (FDMI) was calculated by subtracting the supplement intake from total DMI.

### Animal behavior

During a 24-hour period, with two evaluations per month, totaling six evaluations. The evaluations were performed by means of visual observations [11] with intervals of ten minutes, with the help of binoculars and a stopwatch. Lanterns were used for night observations. Nine animals per treatment were observed to assess variables such as access to the trough, rumination, grazing and other activities [12]. Besides these evaluations, observations were made with regards to the number of chews and rate of bites [13], whose value multiplied by the grazing time provided the information on daily number of bites, bite mass, steps taken at 10 stations and the number of feeding stations. The feeding station was considered as the space corresponding to grazing without movement of the front legs [14] and a step was defined as each movement of the front legs. The number of bites per feeding station was calculated by dividing the number of feeding stations by the number of bites. In each evaluation of feeding behavior, the variables of displacement in grazing were measured three times during the morning and three times during the afternoon for each tester animal.

### Nutritive value

By means of the hand-plucking technique [15], samples were obtained for bromatological analyses. The sampled material was partially dried in a forced ventilation oven at 55°C for 72 hours. After drying, the samples were ground in a Wiley mill through a 1-mm sieve and sent to determine the nutrient value (Table 1). The samples were analyzed for DM, ash, organic matter (OM), crude protein (CP) through the *micro-Kjeldahl* method [16], and for neutral detergent fiber (NDF) and acid detergent fiber through the method of Van Soest et al [17]. The IVDMD and *in vitro* organic matter digestibility (IVOMD) were analyzed [10,18], using the Ankom Fiber Analyzer2000 (Ankom Technology, Macedon, NY, USA). Total digestible nutrients (TDN) were estimated following the method [19], using the equation TDN = % OM×[(26.8+0.595×(IVOMD)] /100.

### Statistical analysis

Pasture characteristics and animal performance were submitted to analysis of variance, using the general linear model produce [20], with significance p = 0.05, considering the treatments (T), periods (P), and their interaction (T×P). Means were compared by the Tukey’s test. The behavioral variables were submitted to MIXED produce (mixed models), using the period with random effect and animal with subject [20]. When the effect of treatments was significant, the variables were compared by the Tukey-Kramer. The maximum likelihood estimation, which is an array of variances and covariance that best fits the data, was used considering the corrected Akaike information criterion [21]. We tested the matrices, variance component, unstructured, and autoregressive of first order. We used the SAS (Cary, NC, USA) University edition.

## RESULTS

The interaction T×P was not significant. The bromatological characteristics of the pasture, although not subjected to statistical analysis, were similar between treatments (Table 1).

The FM was different between treatments (Table 2), being greater for steers fed without legume. However, accumulation rate, forage allowance, and SR did not differ between treatments due to the adequate adjustment of forage allowance. Although the SR did not differ, the grass + supplement treatment lead to a LW 256 kg greater than in grass + legume, influencing the LWG per area.

The final weight of animals, as well as the DMI (kg/d), did not differ (p>0.05) between treatments (Table 3). However, forage intake was higher for non-supplemented animals in relation to supplemented steers (Table 3). Supplement intake did not alter (p>0.05) the TDN intake due to pasture quality.

The ADG did not differ between treatments (p>0.05). Animals fed grass + supplement had higher LWG per area than those fed grass + legume. On the other hand, intermediate values were observed for vetch associated with supplementation. The supplementation lead to a 2.13 (grass + supplement) and 1.7 kg (grass + legume + supplement) greater LW ha/d (Table 3). Animals without supplementation spent more time in grazing (Table 4). The treatments not influenced the feeding behavior of steers (Table 5).

## DISCUSSION

The LWG per area is the trait that varied the most among treatments. The LWG per area was influenced by the SR and average daily weight gain. Stocking rate, as well as the average daily gain, were not affected by treatments. However, the higher values observed for SR and animal gain per day in supplemented-animals led to greater (p<0.05) LWG per area. This gain was 47% and 38% higher in grass + supplement and grass legume + supplement treatments, respectively, compared to pastures mixed with legumes without supplement. This represents an additional of 179 kg/ha due to the use of supplement throughout 84 days of grazing. For the adaptation period (16-days), this value was 213 kg/ha.

This result confirms the efficacy of supplementation to increase animal production per unit of area. Peyraud et al [1] state that legumes have low palatability, leading to lower preference by ruminants, which can reduce gains. This may explain the lack of difference in weight gain per day, since the legume, even of greater nutritional quality, may have contributed in small amounts to the diet. In addition, the DMI (kg/d) was not affected by the presence of legume. Despite this, animal gain per day was high due to excellent nutritional quality of pastures (Table 1).

The CP content was always above 12%, the value required for the animal category of the present study [22]. We expected that supplemented animals would have higher individual performance. Besides providing a more energetic diet, supplementation would provide a more adequate TDN:CP ratio, since excess CP could cause a nutritional imbalance due to the energy cost involved to eliminate this excess. This would reduce the efficiency of energy use and might decrease animal performance [23]. Despite such expectation, the TDN:CP ratio found in all treatments was below 7, which, is the limit at which nitrogen excess occurs [2]. In addition, these authors also state that the effect of supplementation on animal gain per day is more evidenced in low quality pastures, such as tropical pastures. However, in the present study, the CP content was 15% greater and the TDN intake with or without supplementation was similar due the high nutritive value of the pasture. This shows that temperate pastures lead to a substitute effect when supplementation is used.

The DMI did not indicate an additive effect of supplementation since it was similar among treatments. Similarly, the number of bites and bite mass did not differ. The most important variable relative to intake is the bite mass [24]. This depends on forage allowance and quality, which was similar among treatments in the present study. The number of bites per station, number of stations and displacement rate also behaved in a similar way, leading to the similar DMI.

There was occurrence of a substitutive effect. The substitutive effect is expressed when the animal stops consuming forage to consume supplements by selecting forage species or parts of plants (leaves) that are more nutritious. The forage intake was 50% lower for supplemented-animals (p>0.05), consequence of the decrease in time spent in grazing (Table 4). Non-supplemented steers grazed for longer times than supplemented animals, agreeing with the findings [25]. This is influenced by the lower forage mass (Table 2), but this did not influence the animal gain per day since the DMI and TDN intake was similar between treatments.

The grazing time was lower in supplemented-steers compared to those without supplementation (Table 4) because part of the animal’s requirement was supplied by the supplement, while non-supplemented animals need to meet their nutritional requirements consuming forage.

Although the grazing time was similar with or without vetch (p>0.05), no differences were observed in animal gain per day, even though its greater nutritional value. Although the botanical composition was not evaluated, a low proportion of legume was observed, which may be a possible explanation for the similar gain. Studying oat-ryegrass with different seeding densities of vetch (0, 15, 30, and 45 kg/ha), when the proportion of vetch was only 8%, animal gain per day was similar compared to the treatment without legume [26]. On the other hand, when the proportion of vetch was increased to 12%, animal gain per day increased from 0.698 to 0.814 kg/d.

The steers ruminated for around 298.86 min/d, regardless of the treatment (Table 4). The rumination time is influenced by the nutritional quality of the pasture, which behaved similarly. Still, the rumination time is influenced by the type of diet and the cell wall thickness of forage [27]. The NDF content was around 48% for the different treatments, not interfering with rumination or the number of chewing movements (Table 5), which are highly influenced by the cell wall constitution.

The forage mass for the grass-legume treatment did not affect animal gain per day. In the treatment mixed with vetch and without supplementation, forage mass was lower, but it was not enough to influence the weight gain due to the good quality of the pasture. In addition, the criterion for pasture management was forage allowance, which remained above 0.95 kg DM/kg LW/d and did not differ between treatments. This allowed selective grazing and, probably, it did not limit forage intake. This trait showed no difference, since the forage allowance was 3 to 4 times greater than the forage intake.

Competition between species [28] is one of the factors related to lower forage mass when legumes are used. Vetch, as well as ryegrass, has a late cycle, which may have impaired the yield of both species. Plants with high initial growth rate more efficiently use the available resources [29]. Forage suffered from water stress, first with the excessive rainfall in June (over 300 mm) and then in August the opposite happened, and rainfall was only 25 mm (Figure 1). It may have limited vetch production, which is more sensitive to this situation.

According to Peyraud et al [1], the decrease in pasture quality with the advancement in plant age is greater in grasses compared to legumes. This could result in improved response of legume, leading to a higher individual performance at the end of the pasture cycle as a result of the better nutritional value of legume.

The early termination of the study due to the water stress may also have adversely affected treatments with legumes. It is noteworthy that legumes have bacteria that fix nitrogen from the atmosphere, which reduces the use of chemical fertilizers, generating an indirect gain for the system and making it more sustainable [30]. The lack of difference in SR (Table 2) and average daily gain demonstrate that the use of vetch without supplement allowed great animal performance, that is, the presence of legume even in a reduced proportion in grazing systems could make the system more sustainable for diversification of pasture ecosystems.

The supplementation in temperate pasture was shown to be a better alternative for backgrounding beef cattle, since in this category there is no need for extreme gains, in addition to the greater gain per area. However, the long-term use of legumes can benefit the system, considering the lower inputs of nitrogen fertilizer, leading to economic and environmental benefits.

## CONCLUSION

Temperate grasses mixed with or without vetch of great nutritive value allowed an animal gain per day similar to the energy supplementation. Behavioral activities and feeding behavior are similar when pasture management is adequate.

Steers receiving supplementation had a substitutive effect, leading to greater animal gain per area. The association between grasses and supplementation, without mixed with vetch, raises the gain per area. The use of legume without supplement increases the time spent in grazing.

## Figures and Tables

**Table 1:** Nutritive value of ingredients consumed by beef steers in oat and ryegrass pastures mixed or not with vetch and with or without energy supplementation

Component (%)	Treatments			Corn

	Grass+supplement1)	Grass+legume+supplement2)	Grass+legume3)	
Dry matter	94.10	93.95	93.95	88.64
Organic matter	85.3.6	83.73	84.38	84.99
Neutral detergent fiber	48.16	49.65	48.25	22.89
Acid detergent fiber	28.60	27.00	28.37	3.78
Lignin	2.42	2.78	2.74	5.75
Ether extract	2.28	2.14	1.83	2.01
Total digestible nutrients	66.42	64.65	63.96	80.94
Crude protein	15.93	18.22	17.46	7.71
Total digestible nutrients:crude protein4)	4.17	3.54	3.66	10.5
*In vitro* dry matter digestibility	81.81	83.04	81.22	85.98
*In vitro* organic matter digestibility	73.55	73.44	72.23	82.74

1) Oats+ryegrass+supplementation.

2) Oats+ryegrass+vetch+supplementation.

3) Oats+ryegrass+vetch.

4) Relation between total digestive nutrients and crude protein (kg/kg).

**Table 2:** Forage mass, forage allowance, accumulation rate and stocking rate in oat and ryegrass pastures mixed or not with vetch and with or without energy supplementation

Variables	Treatments			Standard error	p-value

	Grass+supplement1)	Grass+legume+supplement2)	Grass+legume3)		
Forage mass (kg DM/ha)	1,487.4^a^	1,303.5^ab^	1,108.7^b^	132.47	0.0355
Forage allowance (kg DM/kg LW)	1.07	0.99	0,96	0.15	0.69
Accumulation rate (kg DM/ha/d)	64.1	65.8	63.0	8.19	0.9178
Stocking rate (kg/ha)	1,411.0	1,313.7	1,155.0	111.33	0.0773

DM, dry matter; LW, live weight.

1) Oats+ryegrass+supplementation.

2) Oats+ryegrass+vetch+supplementation.

3) Oats+ryegrass+vetch.

ab Different letters, in the row, differ statistically (p<0.05).

**Table 3:** Averages for initial and final weight, dry matter intake, average daily gain and live weight gain per area of beef steers in oat and ryegrass pastures mixed or not with vetch and with or without energy supplementation

Variables	Treatments			Standard error	p-value

	Grass+supplement1)	Grass+legume+supplement2)	Grass+legume3)		
Initial weight (kg)	181.3	193.7	206.3	9.98	0.0691
Final weight (kg)	275.5	291.2	286.4	14.73	0.4549
Total intake (kg/d)	7.97	7.83	8.41	0.369	0.2111
Forage intake (kg/d)	5.69^b^	5.45^b^	8.41^a^	0.332	<0.0001
Total digestible nutrients intake (kg/d)	5.63	5.45	5.37	0.27	0.2101
Average daily gain (kg/an/d)	1.12	1.16	0.95	0.138	0.231
Live weight gain (kg/d/ha)	6.59^a^	6.16^ab^	4.46^b^	0.818	0.0412

1) Oats+ryegrass+supplementation.

2) Oats+ryegrass+vetch+supplementation.

3) Oats+ryegrass+vetch.

ab Different letters, in the row, differ statistically (p<0.05).

**Table 4:** Time (minutes) spent on behavioral activities by beef steers in oat and ryegrass pastures mixed or not with vetch and with or without energy supplementation, in the 24-hour period

Variables	Treatments			Standard error	p-value

	Grass+supplement1)	Grass+legume+supplement2)	Grass+legume3)		
Other activities	648.93	655.46	542.64	58.84	0.3196
Grazing	479.68^b^	451.31^b^	580.83^a^	31.62	0.0126
Rumination	277.62	303.26	315.69	26.56	0.7058
Access to the trough	33.76	29.63	-	2.12	0.3576

1) Oats+ryegrass+supplementation.

2) Oats+ryegrass+vetch+supplementation.

3) Oats+ryegrass+vetch.

ab Different letters, in the row, differ statistically (p<0.05).

**Table 5:** Feeding behavior of beef steers in oat and ryegrass pastures mixed or not with vetch and with or without energy supplementation

Variables	Treatments			Standard error	p-value

	Grass+supplement1)	Grass+legume+supplement2)	Grass+legume3)		
N chews (min)	66.6	66.48	71.22	2.08	0.1893
N bites (bites/min)	39.16	42.03	40,79	2.71	0.8149
N bites	18,341	18,982	23,599	2,398	0.2510
Bite mass (g dry matter/bite)	0.32	0.30	0.36	0.067	0.4920
Steps at 10 stations	36.82	35.92	38.05	2.80	0.8504
N of stations	1,599.76	1,632.15	1,867.39	116.94	0.2171
Steps (steps/min)	12.71	13.43	12.36	1.5906	0.8904
Bites per station	12.33	11.92	13.22	2.05	0.8996

1) Oats+ryegrass+supplementation.

2) Oats+ryegrass+vetch+supplementation.

3) Oats+ryegrass+vetch.
